# Molecular Mechanisms and Therapeutic Effects of (−)-Epicatechin and Other Polyphenols in Cancer, Inflammation, Diabetes, and Neurodegeneration

**DOI:** 10.1155/2015/181260

**Published:** 2015-06-09

**Authors:** Joseph Shay, Hosam A. Elbaz, Icksoo Lee, Steven P. Zielske, Moh H. Malek, Maik Hüttemann

**Affiliations:** ^1^Center for Molecular Medicine and Genetics, Wayne State University, Detroit, MI 48201, USA; ^2^Karmanos Cancer Institute, Detroit, MI 48201, USA; ^3^Department of Radiation Oncology, Wayne State University, Detroit, MI 48201, USA; ^4^College of Medicine, Dankook University, Cheonan-si, Chungcheongnam-do 330-714, Republic of Korea; ^5^Integrative Physiology of Exercise Laboratory, Physical Therapy Program, Department of Health Care Sciences, Wayne State University, Detroit, MI 48201, USA; ^6^Cardiovascular Research Institute, Wayne State University, Detroit, MI 48201, USA

## Abstract

With recent insight into the mechanisms involved in diseases, such as cardiovascular disease, cancer, stroke, neurodegenerative diseases, and diabetes, more efficient modes of treatment are now being assessed. Traditional medicine including the use of natural products is widely practiced around the world, assuming that certain natural products contain the healing properties that may in fact have a preventative role in many of the diseases plaguing the human population. This paper reviews the biological effects of a group of natural compounds called polyphenols, including apigenin, epigallocatechin gallate, genistein, and (−)-epicatechin, with a focus on the latter. (−)-Epicatechin has several unique features responsible for a variety of its effects. One of these is its ability to interact with and neutralize reactive oxygen species (ROS) in the cell. (−)-Epicatechin also modulates cell signaling including the MAP kinase pathway, which is involved in cell proliferation. Mutations in this pathway are often associated with malignancies, and the use of (−)-epicatechin holds promise as a preventative agent and as an adjunct for chemotherapy and radiation therapy to improve outcome. This paper discusses the potential of some phenolic compounds to maintain, protect, and possibly reinstate health.

## 1. Introduction: Structural Characteristics of Polyphenols

Polyphenols belong to a ubiquitous family of naturally occurring compounds that encompass several other classes of compounds such as flavonoids. Flavonoids consist of several groups of compounds called anthocyanins, flavanols, flavonones, flavones, and isoflavones. These compounds are polyphenols due to the presence of multiple phenolic units in their chemical structure. Thus, phenolic compounds share structural features including an aromatic or a phenolic ring. Polyphenol compounds are most abundant in fruits, vegetables, cereals, and beverages. Fruits such as apples, grapes, pears, cherries, and berries contain 200–300 mg of polyphenols per 100 grams [1]. (−)-Epicatechin, the focus of this review article, belongs to the group of flavanols. It is most commonly found as a natural product in cacao and cacao products, such as dark chocolate, and in green tea.

## 2. Biological Functions

Polyphenols have various important biological properties in both plants and animals that can be divided into two main categories, with antioxidant and nonantioxidant function. These functions are discussed throughout this paper. Regarding antioxidant action, it is noteworthy that polyphenols are the most abundant antioxidants in the diet with a total daily intake as high as 1 gram, exceeding the intake of vitamin C by about 10-fold and that of vitamin E and carotenoids by about 100-fold [2]. Given the large number of studies showing beneficial effects with vitamin antioxidants, similar or better effects might be expected for polyphenols. Antioxidants, in general, have been intensely studied due to the high prevalence of oxidative stress found in numerous disease states, including Alzheimer's disease, muscular dystrophy, rheumatoid arthritis, diabetes, cancer, heart disease, and aging. For example, in a randomized clinical trial for Alzheimer's disease (AD), patients were treated for 16 weeks with vitamin E (*α*-tocopherol/E) 800 IU daily, 500 mg of vitamin C daily, 900 mg of *α*-lipoic acid (ALA) daily, and 400 mg of coenzyme Q (CoQ) three times daily or placebo [3]. The study showed, following E/C/ALA treatment only, a 19% decrease in F2-isoprostanes, which are cerebral spinal fluid (CSF) biomarkers of AD [3], suggesting the potential application of antioxidant treatment in patients with AD. Oxidative stress has also been found to play a pivotal role in the development of complications due to diabetes, such as cardiovascular and microvascular disease. Following treatment of diabetic mice with vitamins C, E, and *β*-carotene for 8 weeks Mekinová et al. [4] observed reductions of thiobarbituric acid reactive substances (TBARS, used to determine oxidative stress status), glutathione, and glutathione peroxidase and an increase in copper and zinc superoxide dismutase (CuZn-SOD). These examples all argue for the potential use of ROS scavengers including natural compounds with such activities in certain diseases where the redox balance and ROS load are not any longer under control, a research direction that should be pursued with polyphenol compounds in the future.

There are numerous nonantioxidant functions of polyphenols with select examples discussed later in this paper. These include effects on estrogen receptor activity, cell signaling cascades, and cell cycle control in mammalian cells. Since polyphenols are plant-derived compounds, it is not surprising that they play important roles in plant physiology. As an example related to plant signaling, flavonoids were found to greatly affect the growth pattern of* Malus x domestica*, the apple tree [5]. The authors found, following RNAi silencing of the enzyme chalcone synthase (CHS), which is responsible for flavonoid synthesis in apples, a loss in skin and leaf pigmentation and a reduction in size, with smaller leaves and shortened internode lengths [5]. This suggests that flavonoid production is important for the integrity and morphology of apples. Polyphenols also have the ability to scavenge reactive oxygen species (ROS). This is thought to be a primary function of polyphenols in mammals and therefore they are typically referred to as antioxidants.

## 3. Beneficial Health Effects of Selected Flavonoid Compounds

In this section we will briefly summarize the cellular and organismal effects of the selected flavonoids epigallocatechin gallate, genistein, apigenin, and (−)-epicatechin, the latter of which will be discussed in more detail.

### 3.1. Epigallocatechin Gallate

**Figure 1:**
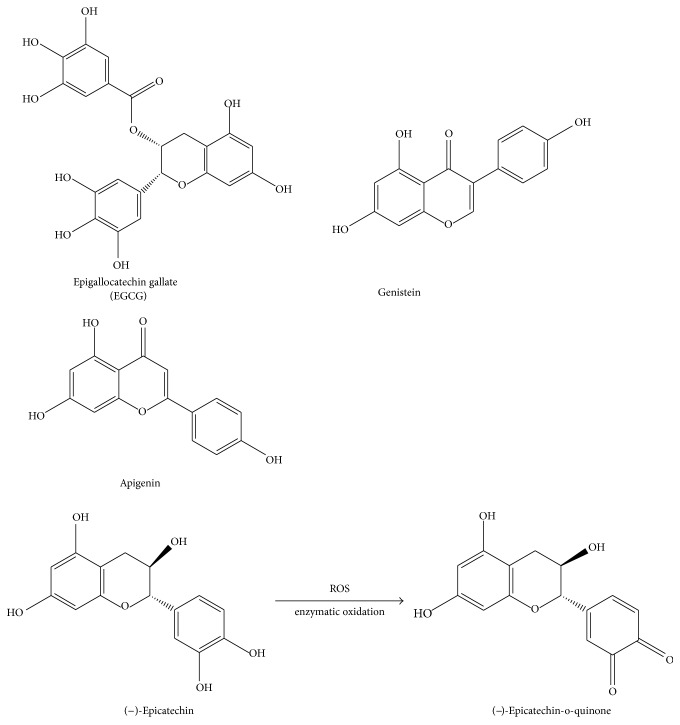
Chemical structures of epigallocatechin gallate, genistein, apigenin, and (−)-epicatechin and its oxidation product (−)-epicatechin-o-quinone.

Epigallocatechin gallate (EGCG) (Figure 1) is the most abundant catechin found in green tea (*Camellia sinensis*) [6]. EGCG is a potent antioxidant that has various clinical applications. It is a widely studied catechin in cancer research and the potential underlying mechanisms have started to emerge. As an example, Lin et al. [7] demonstrated that treatment with EGCG inactivated the STAT3 pathway, which plays a critical role in promoting tumor formation in tumor initiating cells of nasopharyngeal carcinoma. The study showed a reduction in the stemness of tumor initiating cells by sphere formation, colony formation, cell viability, and an increased sensitivity to cisplatin, indicating that the compound directly affects growth signaling in cancer cells. Mukherjee et al. [8] reported that EGCG is able to sequester the p65 subunit of the transcription factor NF-*κ*B and to inhibit cytokine and chemokine transcription following CpG synthetic oligodeoxynucleotide treatment in DU145, PC3, and LNCaP prostate cancer cell lines. This suggests that EGCG is able to ameliorate chronic inflammation resulting from microbial pathogens that increases the risk for prostate cancer. Poutahidis et al. [9] observed that* Apc* ^*Min/+*^ mutant mice, upon gastrointestinal tract infection with* Helicobacter hepaticus*, were significantly predisposed to prostate cancer, suggesting that infection-mediated inflammation can drive cancer progression. EGCG was further found to decrease protein expression of both HIF-1*α* and its downstream target vascular endothelial growth factor (VEGF) in MCF-7 cells in a dose-dependent manner [10]. In addition, EGCG was found to protect the cells from ionizing radiation. A recent study showed that, with a simple pretreatment of 50 *μ*M EGCG, human epidermal keratinocytes (HaCaT cell line) were protected from radiation-induced (20 Gy) cytotoxicity [11]. This was demonstrated by a reduction in (1) apoptotic cells as analyzed by flow cytometry of annexin V/propidium iodide double staining, (2) damage to the mitochondria analyzed by MitoTracker Red staining and upregulation of superoxide dismutase 2, (3) total cellular ROS as determined by DCFDA staining, and (4) *γ*H2AX foci, a marker of DNA damage [11]. They also observed an EGCG-dependent transcriptional induction of heme oxygenase-1, which the authors concluded to be the primary source of the protective effect. After treatment with EGCG following either siRNA knockdown or use of a specific inhibitor of heme oxygenase-1, the protective effects diminished [11].

Epidemiological and animal studies further indicate that EGCG shows protective activity in neurological disorders [12]. In vitro, EGCG was shown to inhibit the aggregation of amyloidogenic proteins including amyloid-*β* (A*β*) monomers, *α*-syn, calcitonin, hIAPP, and insulin, which are important in many neurological diseases [13–16]. Interestingly, in the presence of EGCG, A*β* monomers adopt a new conformation with an increased inter-center-of-mass distance with reduced *β*-sheet content [17]. This thereby affects its inclination to form fibril-prone states that otherwise increase the severity of Alzheimer's disease [17]. Taken together, these studies indicate that EGCG has multiple cellular effects, is able to decrease the risk of tumor initiation and progression, and may be useful in preventing amyloid formation seen in neurodegenerative diseases.

### 3.2. Genistein

Genistein (Figure 1) belongs to the group of isoflavones and is found primarily in soybean seeds [18]. It is also a common precursor in legumes derived from the biosynthesis of antimicrobial phytoalexins and phytoanticipins [18]. Genistein may be useful in the treatment of breast cancer due to its estrogen receptor antagonist activity. It shares a structural similarity with 17*β*-estradiol (estrogen), allowing it to specifically interact with the estrogen receptor. Chen and Chien [19] treated malignant human breast cancer MCF-7 cells with the phytoestrogens genistein, resveratrol, and quercetin and found that they were all individually effective at inhibiting cancer cell growth at concentrations of 10^−4^ M. Furthermore, there was a significant increase in apoptotic MCF-7 cells observed following the treatment with the three compounds, which was primarily due to a reduction in PI3K and Akt phosphorylation and an increase in Fas ligand, Fas-associated protein with death domain (FADD), truncated Bid, cytochrome* c* release, and caspases 3 and 9 activation [19]. Interestingly, when the authors treated the normal MCF-10A cell line with genistein, resveratrol, and quercetin, they observed a slight increase in cell proliferation, which was a result of an increase in PI3K and Akt phosphorylation [19], suggesting that it exerts protective effects on normal tissue. The Jordan group [20] went further and looked at the efficacy of phytoestrogens as a natural alternative to hormone replacement therapy using MCF-7 breast adenocarcinoma cells. They looked at both phytoestrogens and steroidal estrogens (17*β* estradiol and equilin) in fully estrogenized MCF-7 cells and long term deprived MCF-7:5C cells. Both steroidal estrogens and phytoestrogens were found to induce proliferation of MCF-7 cells while they inhibited growth and induced apoptosis in MCF-7:5C cells [20]. This effect was abated when siRNA targeted to estrogen receptor *α* was used, indicating that this is a direct effect of the estrogen receptor. In addition, phytoestrogens were found to induce endoplasmic reticulum stress (via* DDIT3*,* IRE1α*, and *α*-eIF2*α*) and inflammatory response stress (via caspase-4,* CEBPβ*,* IL6*, and lymphotoxin-*β*) in MCF-7:5C cells [20]. Using the corticosteroid dexamethasone in order to inhibit inflammation, the induction of apoptosis and growth inhibition was also blocked [20], suggesting that phytoestrogens may be useful as chemopreventive compounds in patients and postmenopausal women. However, anti-inflammatory agents have antiapoptotic effects, which should be factored into the decision-making process for the treatment plan in the context of cancer therapy.

Genistein was shown to have a 20-fold higher binding affinity to estrogen receptor *β* than estrogen receptor *α* [21]. It also is 130-fold more potent than its counterpart 17*β*-estradiol (estrogen) to bind to estrogen receptor *α* [21]. Estrogen is commonly prescribed to postmenopausal women and is approved for the treatment of osteoporosis. Estrogen deficiency causes both early and late stages of osteoporosis in postmenopausal women by increasing osteoclast formation and activity [22]. In a randomized trial, hormone treatment with 0.625 mg/day of conjugated equine estrogen resulted in a 16.1% decrease in fasting insulin levels and 2.2 mg/dL lower fasting glucose level [23]. For these reasons, genistein has been explored and found to play a protective role against diabetes. For example, genistein was administered at 2, 4, and 6 mg/kg to female nonobese diabetic (NOD) insulin-dependent susceptibility 3 (Idd3) mice, which are predisposed to type 1 diabetes and maintained on a soy and alfalfa-free diet [24]. The mice treated with 2 mg/kg of genistein had a 55–79% decreased incidence of type 1 diabetes starting at 14 weeks after exposure. This effect, however, was not sustainable after 23 weeks. The two higher dose treatments had a significant decrease in incidence starting at 16 weeks [24], suggesting that the most effective dose of genistein based on this study was 2 mg/kg. The compound has also been studied in the context of preventative care of diabetes. Methylglyoxal is considered a major precursor to the so-called advanced glycation end products, which are believed to be one of the major causes of diabetes and its complications, and genistein was found to directly scavenge and thus neutralize methylglyoxal [25]. Finally, in a randomized clinical trial, at 1 year genistein treatment was found to reduce the fasting glucose and fasting insulin levels and insulin resistance compared to the placebo recipients, whose levels remained unchanged [26]. Genistein was also found to increase HDL and to lower LDL, triglycerides, the adipocyte hormone visfatin, and homocysteine blood levels [26].

### 3.3. Apigenin

Apigenin (Figure 1) is a flavone and is commonly found in Chinese cabbage (187 mg/kg), bell pepper (272 mg/kg), garlic (217 mg/kg), bilimbi fruit (458 mg/kg), French peas (176 kg/mg), guava (579 mg/kg), wolfberry leaves (547 mg/kg), and celery (339 mg/kg) [27]. It has been suggested in recent studies to be useful in the treatment of skin and colon cancer. According to the Birt group [28], treatment with up to 80 *μ*M of apigenin caused a time- and dose-dependent cell cycle arrest in G2/M phase. These studies were performed on SW480, HT-29, and Caco-2 colon carcinoma cell lines. After 48 hours following treatment with 80 *μ*M of apigenin, 64%, 42%, and 26% of SW480, HT-29, and Caco-2 cells were arrested, respectively, in contrast to only 15% of the control cells. By immune complex kinase assay, p34 (cdc2), which is a critical enzyme in the G2/M transition, was found to be inhibited in all three cell lines. Western analyses confirmed these findings showing a decrease in expression of both p34 and cyclin B1 proteins. This effect was shown to be reversible when apigenin treatment was discontinued [28]. Apigenin was also explored in breast cancer progression. An epidemiological study illustrated that apigenin at low doses (10–50 *μ*M) was able to cause a dramatic reduction in DNA synthesis after 24 hours in all breast cancer cell lines tested (MDA-MB-468, MDA-MB-231, MCF-7, and SK-BR-3) [29]. However, the viability of these cell lines remained unchanged. Flow cytometry with Oregon Green/PI staining showed that apigenin at a concentration of 30 *μ*M had a cytostatic effect by arresting the cells in G2/M phase [29]. Apigenin was further studied for a potential benefit to the immune system. Warat and colleagues found it to inhibit the expression of the TRAIL-R1 death receptor in RAW264.7 macrophages [30]. These studies suggest at least two significant roles that apigenin can play in antitumorigenesis, by inhibiting cell proliferation and by improving immune cell survival.

### 3.4. (−)-Epicatechin

The Kuna Indians, indigenous people living on islands near the coast of Panama, consume large amounts of cocoa on a daily basis [31]. They have low blood pressure and a significantly lower incidence of cardiovascular disease [32]. There is strong evidence that continuous cocoa consumption and not genetic differences causes the effect, since it is lost when cocoa consumption is discontinued. Unfermented cocoa beans contain 120–180 g/kg of polyphenols with (−)-epicatechin being the main polyphenolic compound approximating 35% [33]. Given its high abundance it is likely that (−)-epicatechin is a key mediator of the beneficial effects of cocoa. A short-term study with healthy humans who received high-flavonoid dark chocolate containing 46 mg (−)-epicatechin daily for 2 weeks showed superior vascular function as seen by improved endothelium-dependent flow-mediated dilation of the brachial artery [34]. However, there were no measurable beneficial short-term effects on other parameters including blood pressure and lipid parameters, suggesting that continued uptake is required to achieve a higher impact on the cardiovascular system as seen in the Kuna Indians. (−)-Epicatechin has also been identified as an important bioactive compound in* Pterocarpus marsupium*, a tree that is widely distributed in central, western, and southern regions of India and used as an important traditional medication in India for the treatment of diabetes and other pathologies including those of the heart and liver (reviewed in [35]). The well-established benefits of* Pterocarpus marsupium* extract as a therapy for diabetes are likely due to the insulin-mimetic effects of (−)-epicatechin [36, 37].

Structurally, (−)-epicatechin is comprised of two aromatic rings linked by an oxygenated heterocycle with a 4-hydroxyl group (Figure 1). It is a compound with high bioactivity when analyzed in isolation. When taken orally, flavanols including (−)-epicatechin are stable during gastric transit but become glucuronidated and partially methylated in the small intestines, processes that continue in the liver, leaving only smaller levels of native (−)-epicatechin in the mesenteric circulation (reviewed in [38]). A small quantitative clinical study with human subjects consuming 80 grams of procyanidin-rich chocolate containing 137 mg (470 *μ*mol) (−)-epicatechin showed that blood (−)-epicatechin increased 12-fold over baseline levels to 257 ± 66 nmol/L after 2 hours and then declined to baseline levels in 8 out of the ten subjects after 6 hours, while it further increased in the remaining two individuals [39]. This suggests that there is a large heterogeneity regarding the half-life and metabolism of (−)-epicatechin in humans. Bioavailability of native (−)-epicatechin is therefore smaller than for vitamins C and E, with about ~1/200 and ~1/150 bioavailability, respectively [39]. Given that most of the ingested (−)-epicatechin undergoes chemical modifications, the glucuronidated and methylated products likely play a key role for the biological effect in addition to the native compound.

#### 3.4.1. Reactive Oxygen Species and Redox Balance

There is a large body of literature proposing that one of the main beneficial effects of (−)-epicatechin is via its ability to directly or indirectly scavenge ROS by chemically reacting with ROS or by modulating pathways that regulate ROS scavenging compounds and enzymes, respectively. Jung et al. [40] identified hydroxyl groups as the crucial structural feature of flavonoids responsible for ROS scavenging. They analyzed seven flavonoids including kaempferol, kaempferol-7-O-*β*-D-glucoside, (+)-catechin, dihydrokaempferol, hesperetin-5-O-*β*-D-glucoside, naringenin, and 7-O-*β*-D-glucoside and concluded that the inhibitory strength of these compounds on total ROS was increased by the number of hydroxyl groups present. Furthermore, the presence of ortho-hydroxyl groups was essential [40], as seen in the ortho- (o-) catechol moiety in (−)-epicatechin (Figure 1). In support of this concept, a recent study confirmed that the o-catechol moiety of (−)-epicatechin is essential for the direct detoxifying effects in the reaction with superoxide and hydrogen peroxide [41]. The simplest reaction product would be (−)-epicatechin-o-quinone (Figure 1) which can undergo further reactions. Interestingly, this o-quinone product is also generated via enzymatic conversion by peroxidases including myeloperoxidase, and a recent study revealed that this enzymatic reaction causes a strong inhibitory effect of (−)-epicatechin-o-quinone on macrophage migration inhibitory factor (MMIF), a key molecule for promotion and maintenance of the inflammatory response [42]. Using liquid chromatography mass spectrometry the authors found that (−)-epicatechin-o-quinone specifically reacts with the N-terminal proline residue of MMIF leading to inactivation of the protein. The authors proposed that this mechanism could explain the beneficial anti-inflammatory effects of (−)-epicatechin when taken during inflammation. It should be kept in mind that the beneficial effects of (−)-epicatechin in the pathophysiological conditions discussed below may at least in part be explained by the ROS modulating capabilities of the compound.

#### 3.4.2. Inflammation

In addition to acute inflammation as seen in sepsis, many other more chronic diseases including diabetes and cancer have a very important inflammatory component. A central aspect of the pathogenesis of diabetes is mediated via interleukin-1*β* (IL-1*β*), which is released by infiltrating inflammatory cells in the pancreas in type I diabetes. IL-1*β* and other cytokines induce upregulation of the inducible form of nitric oxide synthase (iNOS), leading to the production of nitric oxide and downstream *β*-cell damage and death in the pancreatic islets and thus type I diabetes [43]. (−)-Epicatechin at concentrations of 0.1–1 mM was found to inhibit nitrite formation, a downstream product of nitric oxide, in a dose-dependent manner in the rat *β*-cell line RINm5F and in isolated islets induced with 100 pg/mL of IL-1*β* for 24 hours [43]. The authors showed that (−)-epicatechin inhibited the IL-1*β*-induced expression of iNOS by blocking the nuclear localization of the p65 subunit of NF-*κ*B. In addition, in RINm5F cells, (−)-epicatechin was shown to block the inhibition of insulin release after addition of IL-1*β* [43]. It can be speculated that the antidiabetic effects of genistein (see Section 3.2) and (−)-epicatechin may primarily be a result of their nonantioxidant actions and secondarily of their antioxidant actions. Studies using vitamin E as an antioxidant did not improve outcome except for the group of patients with the haptoglobin 2-2 genotype [44]. However, increased vitamin C plasma levels are a predictive factor for a decreased risk of type II diabetes [45], and vitamin C supplementation in patients receiving metformin improved plasma vitamin C levels as well as fasting and postmeal blood glucose levels [46].

(−)-Epicatechin was also found to be effective in a mouse model of atherosclerosis at daily doses of about 110 mg/kg body weight with an average (−)-epicatechin plasma concentration of 4.2 *μ*M. Such treatment blocked lesion progression in ApoE3-Leiden (E3L) mice being fed an atherogenic Western-type diet (15% cocoa butter, 1% corn oil, 40.5% sucrose, 20% acid casein, 10% corn starch, and 6.2% cellulose, supplemented with 1% cholesterol) for four weeks [47]. Treatment with (−)-epicatechin was found to reduce atherosclerosis by 27% compared to the high calorie diet alone control group. Another study found that (−)-epicatechin treatment improved acute intestinal inflammatory disease. At a concentration of 10 mg/kg in rats induced with trinitrobenzenesulfonic acid, there was a reduction in colitis including less ulceration and disorganization of the tissue after histological analysis [48]. In addition, the authors observed significantly higher levels of glutathione in the colon tissue of animals treated with (−)-epicatechin.

Inflammation is also a central component of allergies [49], which are not typically thought of to be a serious condition. However, there are instances where they can be life threatening, for example during anaphylactic shock. The epidemic rise in allergies over the past few decades has been a major concern, and it is therefore important to explore better therapeutic options. A few recent reports illustrate the attenuating role polyphenols including (−)-epicatechin have in the allergic immune response. Singh et al. [50] studied mice that were sensitized to ovalbumin for 3 days and then challenged weekly with 20 mg of ovalbumin for 7 weeks. The animals were fed with pellets containing 1%, 0.3%, or 0.01% purified (−)-epicatechin for 8 days. After the treatment period the mice were again challenged with 100 mg of ovalbumin after which the authors observed a reduction in many of the clinical symptoms including scratching around the nose or head and diarrhea. They also found a reduction in ovalbumin-specific IgE present in the mice fed with the high dose (26 ± 13 ng/mL) and medium dose (89 ± 35 ng/mL) of (−)-epicatechin compared to control population (144 ± 70 ng/mL) [50]. Another polyphenol family member that is discussed above, EGCG, has also been implicated in ameliorating allergic responses. One study revealed that EGCG directly interacts with ovalbumin leading to a change in the secondary *β*-sheet structure of the allergen, which occurred at a 1 : 1 molar ratio, attenuating the allergic response [51]. These studies indicate that polyphenols including (−)-epicatechin have the capability to reduce inflammatory responses, for example by direct interference with proteins involved in triggering the response.

#### 3.4.3. Cancer, Mitochondrial Metabolism, and Cell Signaling

**Figure 2:**
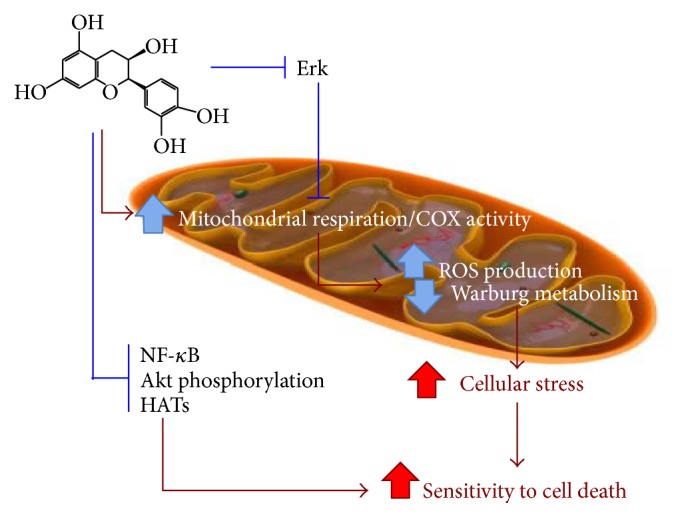
Proposed model of the interference of (−)-epicatechin with cancer signaling, metabolism, and proliferation. (−)-Epicatechin stimulates mitochondrial respiration and biogenesis, thus interfering with Warburg metabolism. At the cell signaling level, the compound inhibits Erk signaling, which interferes with other signaling pathways including EGFR that are known to be hyperactive in cancer. (−)-Epicatechin through Erk and/or other signaling pathways leads to an activation of mitochondrial oxidative phosphorylation, which interferes with Warburg metabolism. Other targets that are inhibited by (−)-epicatechin in cancer cells are NF-*κ*B, Akt, and histone acetyltransferases (HATs). As a result, (−)-epicatechin interferes with cancer signaling, thus rendering the cells more susceptible to apoptosis, an effect that could be utilized to sensitize cancer cells to radiation treatment or chemotherapy. It should be noted that (−)-epicatechin exerts a distinct protective response in noncancerous normal tissue (not shown). This highlights the importance not to generalize the effects but to include detailed information including cell type and treatment regimen.

Inflammation has been linked to cancer development, progression, invasion, and metastasis [52] and the use of anti-inflammatory agents has been proposed as an attractive adjunct therapy for the clinic in the future [53], and we here propose that application of (−)-epicatechin may be such a viable approach. In addition to its anti-inflammatory effect several studies concluded a potential for (−)-epicatechin as a novel anticancer drug mediated through additional mechanisms (Figure 2). (−)-Epicatechin was shown to cause DNA damage and apoptosis in acute myeloid leukemia cells in rats when administered orally at a dose of 40 mg/kg body weight for 22 consecutive days [54]. Additionally, (−)-epicatechin was shown to inhibit the proliferation of Hodgkin's lymphoma cells and Jurkat T cells, which was attributed to the ability of (−)-epicatechin to inhibit the binding of NF-*κ*B to DNA in these cells [55] (Figure 2). Interestingly, these effects were not associated with (−)-epicatechin's antioxidant activity, nuclear translocation of NF-*κ*B, or p65 phosphorylation. The mechanism by which (−)-epicatechin inhibits NF-*κ*B–DNA binding is still open to investigation. One molecular target of (−)-epicatechin that may in part explain the anticancer activity has been identified. It is the Na^+^/H^+^ exchanger, which is strongly inhibited by (−)-epicatechin, and it was proposed that cancer cell plasma membrane fluidity and cytosolic pH are disturbed, thus interfering with cell proliferation [56, 57].

(−)-Epicatechin was also shown to inhibit prostate cancer cell proliferation, potentially by suppressing agonist-dependent androgen receptor activation and androgen receptor-regulated gene transcription [58]. Inhibition of histone acetyltransferase activity was identified as a potential mechanism for reduced prostate cancer cell viability. However (−)-epicatechin shows a weaker potential for inhibiting histone acetyltransferase activity when compared with its analogs EGCG and epigallocatechin. The study suggested that prostate cancer patients with androgen receptor positive tumors might specifically benefit from (−)-epicatechin and its analogs as therapy enhancing drugs. The study further suggested that (−)-epicatechin and related compounds can exploit the genetic variations that are intrinsic to cancer cells since KRAS mutant pancreatic cancer cells appear to be particularly sensitive to (−)-epicatechin's anticancer activity. Another study found that (−)-epicatechin specifically inhibited KRAS mutant pancreatic cancer cells in vitro and in vivo [59]. Here, (−)-epicatechin treatment reduced KRAS mutant pancreatic cancer cell viability but not that of normal cells, and it also reduced GTP-bound Ras protein levels, Akt phosphorylation, and NF-*κ*B transcriptional activity.

The anticancer activity of (−)-epicatechin surpasses the mere inhibition of cancer cell proliferation into prevention of tumor promotion and development. In a mouse model for papilloma formation papilloma was induced with the carcinogen 7,12-dimethylbenz[a]anthracene and promoted using croton oil [60]. The authors showed that topical application of 100 mg (−)-epicatechin/kg body weight inhibited papilloma formation in papilloma-induced mice [60]. Furthermore, oral administration of the same dose of (−)-epicatechin to mice significantly reduced soft tissue fibrosarcoma that was induced by 20-methylcholanthrene. Interestingly, there is some indication regarding the additive or synergistic effect of certain polyphenols. A recent report suggests that panaxadiol, an active compound derived from steamed ginseng, has a synergistic effect when administered together with (−)-epicatechin [61]. Using human colorectal cancer HCT-116 cells they found that combining 20 *μ*M panaxadiol with 150, 200, or 250 *μ*M (−)-epicatechin resulted in growth inhibition of 51%, 97%, and 95%, respectively. The combination also increased the apoptosis level by 11.9%, 16.6%, and 25.8%, as examined by annexin V/PI staining. Thus, there is strong support for the anticancer activity of (−)-epicatechin and its analogs. However, several questions pertaining to the mechanism by which these compounds mediate their anticancer effect should be addressed in future studies including how exactly (−)-epicatechin inhibits the binding of NF-*κ*B to DNA and Akt phosphorylation.

Patients on cancer therapy may benefit from coadministration of (−)-epicatechin together with their conventional therapy for another reason. Several studies showed that (−)-epicatechin protects normal and nontumorigenic tissues from insults by radiation or chemotherapy [62–69]. For example, pretreatment of adult rats with (−)-epicatechin reduced doxorubicin's neurotoxicity by reducing TNF*α*, iNOS, and NF-*κ*B expression, as well as reducing the total nitrite levels in the brains [66]. (−)-Epicatechin's protective effect extends to other chemotherapeutic drugs such as cisplatin where it was found to inhibit mitochondrial and renal damage in vitro using cultured mouse proximal tubular cells and in vivo in mice pretreated with (−)-epicatechin prior to cisplatin administration [67]. A similar (−)-epicatechin-mediated protective effect was observed in cell lines in vitro and in a zebrafish model in vivo after cisplatin treatment, via blocking of ROS generation [68]. In another recent study, (−)-epicatechin significantly increased clonogenic survival and restored the migration ability of irradiated normal human dermal fibroblasts, in which it also inhibited radiation-induced ROS generation, mitochondrial dysfunction, and cell death [63]. Mechanistically, the authors showed that (−)-epicatechin significantly inhibited p-JNK, p38, and cleaved caspase-3 levels when combined with radiation treatment. The same group earlier reported inhibition of radiation-induced apoptosis in a human keratinocyte line (HaCaT), which is another model for normal human cells [64]. Moreover, rats given (−)-epicatechin orally showed reduction in radiation-induced oral mucositis as indicated by increased oral food intake, weight gain, and increased overall survival rates. This is an important finding suggesting that (−)-epicatechin can alleviate a common side effect of cancer therapy that is painful for the patient and significantly reduces quality of life.

Several studies have suggested the potential antiproliferative role many catechins have by acting upon DNA methyltransferases (DNMTs). These are a family of enzymes that catalyze the covalent transfer of a methyl group to the 5-position of the pyrimidine ring of cytosines predominately located within CpG islands. DNA methylation has many significant effects in the mammalian genome including transcriptional regulation, chromatin structure modulation, X chromosome inactivation, genomic imprinting, and the suppression of repetitive and parasitic DNA sequences for genome stability [70]. Flavonoids such as (−)-epicatechin are metabolized in vivo and the products include methylated, sulphated, and glucuronidated derivatives. In addition to the original flavonoids, these downstream products may also exert biological activities. A recent study showed that 4-O-methyl-epicatechin and 3-O-methyl-epicatechin had antiproliferative functions in MCF-7 breast cancer cells and BxPC-3 pancreatic cancer cells, whereas other derivatives showed limited or no activity [71], suggesting that certain metabolites could be developed as cancer therapeutics. Another recent study showed that (−)-epicatechin has the ability to directly inhibit DNA methyltransferase activity [72]. The authors used a novel electrochemical immunosensing model in order to detect the activity of the prokaryotic DNA methyltransferase M. SssI MTase. The DNA helix structure was formed on the surface of a gold nanoparticle glassy carbon electrode where it was then methylated by M. SssI MTase. Following DNA digestion with the restriction enzyme Hpall, which does not recognize methylated CpG regions, and fragment analysis the authors concluded that epicatechin was inhibitory with an IC_50_ value of 129 *μ*M [72], whereas an earlier study reported a value of 8.4 *μ*M [73].

In cancer cells mitochondrial metabolism and respiration are often suppressed with most solid tumors showing a 25–60% decrease in mitochondrial mass compared to healthy cells [74]. Cancer cells shift from mitochondrial respiration to glycolysis, which is known as the Warburg effect [75, 76]. Mechanistically, there is strong evidence that cancer signaling affects the oxidative phosphorylation machinery, and recent studies showed that all components of the oxidative phosphorylation system can be phosphorylated (reviewed in [77]). Among these are cytochrome* c* (Cyt c) and cytochrome* c* oxidase (COX), which catalyze the terminal and proposed rate-limiting step in the mitochondrial electron transport chain [78, 79]. Multiple phosphorylation sites have been mapped on both enzymes by us and others [80–86]. Based on those phosphorylations that have been studied functionally, we proposed that oxidative phosphorylation is decisively regulated by cell signaling (for recent reviews see [87, 88]). As an example in the context of cancer metabolism, the activated EGF receptor was shown to translocate to the mitochondria where it directly interacts with COX catalytic subunit II leading to COX inhibition [89, 90]. A similar translocation to the mitochondria was also reported for receptor tyrosine kinase ErbB2 in cancer cell lines and cancer specimens [91]. Since the oxidative phosphorylation machinery is suppressed in most cancers it can be speculated that reactivation of mitochondrial function might be a strategy to interfere with cancer proliferation. We recently showed that (−)-epicatechin stimulates mitochondrial respiration in vitro in pancreatic cancer cells [69] and in vivo in normal mouse muscle tissues, in which it also significantly stimulated the expression of oxidative phosphorylation protein complexes [92]. Interestingly, (−)-epicatechin treatment sensitized Panc-1, U87, and MIA PaCa-2 pancreatic cancer cells to radiation treatment, significantly reducing clonogenic survival, but it had a small protective effect in normal control cells [69], ideal characteristics for a compound that could be used as an adjunct therapy with radiation or chemo treatment. Another example of the protective effect of (−)-epicatechin on healthy tissue was reported in the context of cisplatin treatment, which can cause nephropathy as a side effect. In mice, (−)-epicatechin, administered 8 hours after renal injury, which was induced by cisplatin treatment, inhibited the decrease in mitochondrial succinate dehydrogenase activity, cytochrome* c* release, mitochondrial fragmentation, and cytochrome* c* oxidase protein levels in the proximal tubular cells [67]. (−)-Epicatechin also exhibited an otoprotective effect in injury induced by cisplatin in a cochlear organ of the Corti-derived cell line HEI-OC1 in vitro and in rats in vivo by inhibiting the activation of ERK, caspase-3, JNK, and the release of cytochrome* c* [62].

The effect of (−)-epicatechin on Erk signaling has been reported by several groups. (−)-Epicatechin was shown to inhibit Erk2 phosphorylation in micromolar concentrations [62, 93]. Erk2 is part of the Ras/MAPK pathway, which is central for several cellular processes including proliferation and survival. It also crosstalks with the EGF pathway [94] and could therefore directly (via phosphorylation of oxidative phosphorylation complexes) or indirectly (via interfering with EGF signaling) regulate mitochondrial activity. Thus in cancer, MAPK pathway hyperactivation by Erk2 phosphorylation could target COX and Cyt c for phosphorylation causing Warburg metabolism. Consequently, inhibiting MAPK pathway activation—with (−)-epicatechin as we propose here—can potentially restore COX and Cyt c activity and mitochondrial respiration. However, the exact mechanism by which the MAPK pathway controls mitochondrial respiration remains to be investigated including the assignment of MAPK pathway-dependent phosphorylation sites on oxidative phosphorylation proteins and their functional effects.

There is clear evidence that (−)-epicatechin affects multiple other signaling pathways and that there are tissue-specific differences in how some of them respond to the compound. (−)-Epicatechin was found to modulate NF-*κ*B, activator protein-1 (AP-1), and nuclear factor erythroid 2p45-related factor-2 (Nrf2) signaling, all being important in cellular detoxification, proliferation, survival, and differentiation [95]. (−)-Epicatechin also reduced p-JNK and p-38 expression in human cultured fibroblasts [63, 64], and it induced phosphorylation of Akt, HSP90, and eNOS in human coronary artery endothelial cells (HCAEC) [96]. In neurons, (−)-epicatechin at 100–300 nanomolar concentration stimulated cAMP-response element binding protein (CREB) phosphorylation [43]. Interestingly, this effect could be plotted on a bell-shaped curve, and at very low concentrations stimulation was not observed and at 30 micromolar concentrations (−)-epicatechin had an inhibitory effect. Such a behavior was also observed for ERK and Akt phosphorylation [43]. Furthermore, (−)-epicatechin promotes vascularization in healthy mouse skeletal muscle via regulation of the expression of angiogenic and antiangiogenic factors, such as VEGF and thrombospondin-1 (TSP-1), respectively [92, 97, 98]. In the same tissue it also stimulates mitochondrial biogenesis through expression of peroxisome-proliferator-activated receptor *γ* coactivator-1*α* (PGC-1*α*), PGC-1*β*, and mitochondrial transcription factor A (TFAM) [98, 99]. Increased mitochondrial biogenesis and function would also counteract the Warburg effect.

Some studies suggested that some of the effects of (−)-epicatechin are mediated through receptors. A recent study explored the use of an (−)-epicatechin-dextran conjugate, which cannot permeate through the cell wall [100]. Their findings show that treatment with both 100 nM (−)-epicatechin and 100 nM (−)-epicatechin-dextran for 10 minutes induced and activated PI3K, PDK-1, Akt, and eNOS in human coronary artery endothelial cells (HCAEC). However, the interesting finding was that (−)-epicatechin-dextran activation was significantly higher than nonconjugated (−)-epicatechin, suggesting the existence of an (−)-epicatechin cell membrane receptor. Panneerselvam et al. concluded that (−)-epicatechin is interacting with cell membrane *δ*-opioid receptors, promoting cardiac protection [101, 102]. In one study, the authors used mice that were treated with either control saline, 1 mg/kg (−)-epicatechin, or 5 mg/kg naltrindole, a *δ*-opioid receptor antagonist, by daily intraperitoneal injection for 10 days [102]. They observed that mitochondria isolated from the hearts of mice treated only with (−)-epicatechin had higher state 3 respiration rates. In contrast, heart mitochondria isolated from mice treated with naltrindole and (−)-epicatechin showed an attenuated state 3 respiration, suggesting that *δ*-opioid receptor activation through (−)-epicatechin augments mitochondrial function [102].

#### 3.4.4. Ischemia/Reperfusion Injury

A pathological condition in which ROS play an essential role is ischemia/reperfusion injury as seen in ischemic stroke and myocardial infarction. These are life-threatening conditions, which are among the top causes of death and long-term disability. They are caused by a restriction of blood flow to either the brain or heart. This process thwarts the transfer of oxygen and nutrients to these sites causing ischemia, which blocks energy production. In order to salvage the affected tissue, blood flow has to be restored, which is referred to as reperfusion. Reperfusion can increase mitochondrial and cellular damage due to excessive mitochondrial ROS production (reviewed in [103]). A key mechanism to ischemia/reperfusion injury that also involves ROS is mitochondrial permeability transition (MPT) priming. When executed, MPT causes mitochondrial membrane leakage, fatty acid accumulation, Cyt c release, antioxidant loss, and changes in intra- and extramitochondrial Ca^2+^ and pH [104]. Given its antioxidant capacity, it is not surprising that (−)-epicatechin has been studied as a cardioprotective therapy. Dose concentrations of 10 mg/kg (injected 15 minutes prior to reperfusion and/or 12 hours later) were found to be cardioprotective in rats, reducing infarct size by 27% after 48 hours and 28% after 3 weeks of reperfusion with a single administration of (−)-epicatechin [105]. Even more substantial effects were seen with the dual application of (−)-epicatechin, with a reduction in infarct size by about 80% at 48 hours and 52% at 3 weeks and improvement of other mitochondrial properties, including oxygen consumption rate and mitochondrial morphology. Protection was also observed when rats were pretreated with 20 mg/kg (−)-epicatechin daily for 21 days following induction of myocardial infarction via isoproterenol injection [106]. (−)-Epicatechin treated rats had reduced serum troponin-I (cTn-I), creatine kinase, and lactate dehydrogenase levels compared to the isoproterenol controls. In isolated heart mitochondria the activities of superoxide dismutase, glutathione peroxidase, and glutathione reductase were all significantly decreased after treatment with isoproterenol [106]. In the (−)-epicatechin pretreated group, these activities were significantly higher, as were activities of mitochondrial marker enzymes, such as succinate dehydrogenase, *α*-ketoglutarate dehydrogenase, and NADH dehydrogenase [106]. Another recent study looked at (−)-epicatechin-specific effects in brain ischemia/reperfusion injury following permanent distal middle cerebral artery occlusion in conjunction with (−)-epicatechin treatment 90 minutes prior to reperfusion [107]. They found a 55%, 40%, and 50% decrease in infarct size in mice treated with 5 mg/kg, 10 mg/kg, and 15 mg/kg (−)-epicatechin, respectively. They next used cultured neurons from wild-type and Nrf2^−/−^ mice. Pretreatment with 50 or 100 *μ*M (−)-epicatechin starting 6 hours prior to oxygen/glucose deprivation protected WT neuronal cultures from oxygen/glucose deprivation, but this was not observed in the neurons isolated from Nrf2^−/−^ mice. This suggests that the protective effects of (−)-epicatechin are mediated through the Nrf2 antioxidant stress response pathway, which was confirmed by the observation that there was a dose-dependent increase in expression of HO-1, FTL, and BVR, which are Nrf2/ARE-regulated proteins [107].

## 4. Conclusion

There is a large body of literature demonstrating that several polyphenols have beneficial health effects and, based on animal models, can be used to treat acute and chronic conditions such as ischemia/reperfusion injury, neurodegeneration, diabetes, and cancer. Several signaling pathways have been implicated including Erk and Nrf2, which regulate proliferation and redox balance, respectively. It is important to note that effects mediated by compounds such as (−)-epicatechin can be distinct or even entirely opposing in different cell types such as cancer cells and normal tissues. Therefore, future work should further dissect the precise mechanisms through which the compounds act including extra- and intracellular sites in healthy and pathological conditions. Such mechanistic knowledge would raise the acceptance and help implement the utilization of these compounds in clinical practice.

## Abbreviations

ALA: *α*-Lipoic acid
CoQ: Coenzyme Q
COX: Cytochrome c oxidase
Cyt c: Cytochrome c
EGC: Epigallocatechin
EGCG: Epigallocatechin-3-gallate
IL-1*β*: Interleukin-1*β*
Nrf22: Nuclear factor erythroid 2p45-related factor-2
ROS: Reactive oxygen species
SOD: Superoxide dismutase.
